# Quantum Dynamics and Kinetics of the F + H_2_ and F + D_2_ Reactions at Low and Ultra-Low Temperatures

**DOI:** 10.3389/fchem.2019.00328

**Published:** 2019-05-14

**Authors:** Dario De Fazio, Vincenzo Aquilanti, Simonetta Cavalli

**Affiliations:** ^1^Istituto di Struttura della Materia, Consiglio Nazionale Delle Ricerche (CNR), Rome, Italy; ^2^Dipartimento di Chimica, Biologia e Biotecnologie, Università degli Studi di Perugia, Perugia, Italy

**Keywords:** scattering resonances, tunnel effect, Wigner threshold law, kinetic isotope effect, cold and ultra-cold collisions

## Abstract

Integral cross sections and rate constants for the prototypical chemical reactions of the fluorine atom with molecular hydrogen and deuterium have been calculated over a wide interval of collision energy and temperature ranging from the sub-thermal (50 K) down to the ultra-cold regimes (0.5 mK). Rigorous close coupling time-independent quantum reactive scattering calculations have been carried out on two potential energy surfaces, differing only at long-range in the reactants' channel. The results show that tunnel, resonance and virtual state effects enhance under-barrier reactivity giving rise to pronounced deviations from the Arrhenius law as temperature is lowered. Within the ultra-cold domain (below 1 mK), the reactivity is governed by virtual state effects and by tunneling through the reaction barrier; in the cold regime (1 mK–1 K), the shape resonances in the entrance channel of the potential energy surface make the quantum tunneling contribution larger so enhancing cross sections and rate constants by about one order of magnitude; at higher temperatures (above 10 K), the tunneling pathway enhanced by the constructive interference between two Feshbach resonances trapped in the reaction exit channel competes with the thermally activated mechanism, as the energy gets closer to the reaction barrier height. The results show that at low temperatures cross sections and rate constants are extremely sensitive to small changes in the long-range intermolecular interaction in the entrance channel of the potential energy surface, as well as to isotopic substitution.

## 1. Introduction

The F + H_2_ reaction has been extensively studied for many years from a variety of perspectives, as reported in numerous papers and reviews appeared in the literature (see e.g., Manolopoulos, 1997; Liu, 2001; Der Chao and Skodje, 2002; Althorpe and Clary, 2003; Qiu et al., 2006; Wang et al., 2018 and references therein). Its peculiarity of being accessible both to theory and to experiments makes it an important prototype to validate methodologies to be used for more complex hydrogen atom transfer reactions, that show up in many areas of chemistry, ranging from processes in space (Neufeld et al., 2005; Goumans and Kästner, 2010) to reactions in biological environments (Nagel and Klinman, 2006). The discovery of the interstellar HF molecule (Neufeld et al., 1997) and its recent observation in several astrophysical environments (see e.g., Emprechtinger et al., 2012 and references therein), has also made this system very appealing for astro-chemistry. The large chemical stability and large dipole moment of the HF molecule make it favorably detectable, so that it may serve as a tracer for molecular hydrogen within the diffuse interstellar medium (Sonnentrucker et al., 2010), a valid alternative to CO molecule, the main tracer of molecular gas. An account of the chemistry of the HF molecule is given in Zhu et al. (2002). Being the F + H_2_ reaction the only source of interstellar hydrogen fluoride, reliable kinetic data at low temperatures are of course highly desirable. In recent years, the field of cold and ultra-cold chemistry has seen a considerable growth becoming a frontier both for applied and theoretical research in physics and chemistry (see e.g., Smith, 2008; Krems et al., 2009; Balakrishnan, 2016). The interested reader is directed to the recent review articles (Herschbach, 2009; Hutzler et al., 2012; Quemener and Julienne, 2012; van de Meerakker et al., 2012) for a detailed description of experimental and theoretical developments of the field of cold and ultra-cold molecules, and to (Lara et al., 2012; Tizniti et al., 2014; Costes and Naulin, 2016) for progress on chemical reactivity at low temperature.

The F + H_2_ reaction and its isotopic counterpart F + D_2_ are exothermic with a low energy barrier along the reaction path connecting reactants to products. At thermal or higher energies these reactions occur as thermally activated process. However, at sub-thermal energies the reaction gains access to quantum phenomena and the reactivity is higher than expected on the basis of classical theories. Quantum effects, such as scattering resonances, i.e., the formation of metastable states of the collision complex, and tunneling through the reaction barrier play a progressively larger role on the reactivity of these systems (see e.g., Althorpe and Clary, 2003 and references therein), as energy or temperature decrease. Quantum threshold effects manifest themselves only at very low temperature, typically 1 K or less, when the de Broglie wavelength becomes comparable to, or longer than, the distances between colliding species (Simbotin et al., 2011, 2014). Theoretical approaches based on classical mechanics fail to describe the under-barrier reactivity, and therefore a detailed understanding of dynamics and kinetics requires quantum mechanical treatments and realistic potential energy surfaces. For a survey of the commonly used scattering theory methods see Schatz (1996).

The analysis of the resonances for the F + H_2_ reaction has been the subject of several previous theoretical and experimental works, for a recent review see Wang et al. (2018). Most of the effort has been devoted to understand the features above 20 meV coming from the large number of the metastable states supported by the van der Waals well in the exit channel (Manolopoulos, 1997; Der Chao and Skodje, 2002; Aquilanti et al., 2004). Nevertheless, it was also evident from experiments and theory (Takayanagi and Kurosaki, 1998; Aquilanti et al., 2005a) that resonances trapped by the van der Waals well in the entrance channel, although probably too narrow to be experimentally resolved, could play some role. In the following we show that the latter dominate the dynamics in the cold energy regime.

In this paper we investigate the dynamics and kinetics of the F + H_2_ and F + D_2_ reactions at low temperatures, in a wide interval extending from near absolute zero to 50 K, where quantum mechanical effects control chemical reactivity. The aim is to determine how large they are and where they show up. In the quantum mechanical low-temperature regime, chemical reactivity is most sensitive to the details of the potential energy surface and small changes in the entrance channel interaction can enhance cross sections and rate constants by orders of magnitude. To this purpose, numerically exact quantum scattering calculations of integral cross sections and rate constants have been carried out on two potential energy surfaces, the Stark and Werner potential energy surface (SW PES hereafter) (Stark and Werner, 1996) and PES-II (Aquilanti et al., 2001, 2005b), differing only in the long-range interaction of the F atom with the H_2_ molecule. Namely, the entrance channel van der Waals well of the PES-II is deeper, wider and shifted to larger intermolecular distances than for the SW PES, see Aquilanti et al. (2005b) for more details.

In the last ten years new *ab-initio* potential energy surfaces have been published for this reaction: they are denoted FXZ (Fu et al., 2008), CSZ (Chen et al., 2015), LWAL (Li et al., 2007; Lique et al., 2011) PESs. Unlike the SW PES, these surfaces include the effect of spin-orbit coupling. However, as more extensively discussed in a previous paper (De Fazio et al., 2016), their reliability in describing the reaction dynamics in the cold and ultra-cold regimes is not as satisfactory as at higher energies. As is known, the neural network algorithm used in the fit of FXZ/CSZ PESs and the splines used to merge the three different local fits of LWAL PES can give rise to significant numerical instabilities when the ab-initio grid points are not dense enough, as they should be to push quantum scattering calculations down to the Wigner limit. The results of some test calculations below 1 K, have shown that none of them met the convergence requirements achieved with SW PES probably because of the artifacts of the fits to the ab initio points, and so we preferred not to present them. As pointed out in reference (Balakrishnan, 2016), the situation for the F + H_2_ system is far from ideal. None of the available PESs provide an accurate treatment of the long-range interaction. As mentioned by the authors of both reference (Chen et al., 2015) and reference (Lique et al., 2011), further refinements in the description of the interaction around the van der Waals well in the entrance channel are needed in order to use CSZ and LWAL PESs in scattering calculations at energy below 0.1 meV.

The choice of PES-II is motivated by the recent experimental measurements (Tizniti et al., 2014) which have confirmed as realistic the description of the van der Waals region in the entrance channel, while the SW PES is used here for purpose of comparison with the results of other theoretical investigations. Further experimental evidences of the reliability of PES-II at low collision energies will be shown in the present article. The SW PES and PES-II have the same barrier height but a slightly different width. Thus, the comparison between the two PESs allows to assess the relevance that barrier width, other than barrier height and exo-ergicity, has on the intermolecular kinetic isotope effect of H-transfer reactions at low temperature.

The first quantum reactive scattering calculations for the F + H_2_ system at ultra-cold temperatures were made more than 15 years ago (Balakrishnan and Dalgarno, 2001; Bodo et al., 2004). However, these papers presented only calculations with zero total angular momentum, so that reliable information was obtained only in the Wigner regime. Convergent rate constants, calculated by the hyper-quantization technique (Aquilanti et al., 1998), down to a few Kelvin, were published by our research group (Aquilanti et al., 2005b). Here, we extend this previous study at very low temperatures where the sensitivity to the entrance channel interaction is larger. Because of the relevance that the analysis of the kinetic isotope effect (KIE) has especially in organic chemistry and biochemistry (see e.g., Roston et al., 2013), the temperature dependence of the intermolecular kinetic isotope effect has also been assessed. The quantum dynamics and kinetics of the F + H_2_ and F + D_2_ reactions are predicted form first principles using a coupled channel method summarized in section 2. The effects of tunneling, resonances and isotope substitution on cross sections and rate constants are discussed in section 3; conclusions follow in section 4.

## 2. Chemical Reactions From First Principles: A Summary of the Theoretical Methodology

Within the quantum mechanical time independent framework for studies of reaction dynamics, we use the Born-Oppenheimer separation of electronic and nuclear motion and solve the Schrödinger equation for the motion of nuclei

(1) $$\begin{array}{l} \left[-\frac{\hbar^{2}}{2\mu }\nabla^{2}+V-E\right]\text{Ψ}=0 \end{array}$$

controlled by the ground electronically adiabatic potential energy surface *V*, with μ being the reduced mass of the system and ∇ denoting the Laplacian operator, applying a convergent close-coupling technique. To describe the concerted bond breaking and bond forming taking place in the hydrogen atom transfer reaction, we use hyper-spherical coordinates: the hyperradius, ρ, playing the role of a reaction coordinate being capable to describe democratically both the reactants and products channels, and five angular variables. Alternative parameterizations of the hyperangles have been proposed, see references (Pack and Parker, 1987; Launay and Le Dourneuf, 1989; Aquilanti et al., 2000). The computer code (Skouteris et al., 2000) used to carry out scattering calculations implements the formalism in Delves hyper-spherical coordinates.

The wave function Ψ is expressed in terms of eigenfunctions of the total angular momentum and internal states of the system. The integration over the angular variables leads to a multichannel scattering problem as a function of ρ

(2) $$\begin{array}{l} \left[\frac{d^{2}}{d\rho^{2}}+\frac{2\mu }{\hbar^{2}}\left(E-\epsilon_{n}\right)\right]F_{nn^{\prime }}\left(\rho \right)-\underset{n^{\prime }}{\sum }W_{nn^{\prime }}F_{nn^{\prime }}\left(\rho \right)=0 \end{array}$$

where ϵ_*n*_ are effective hyperspherical potentials potentials and $W_{nn^{\prime }}$ is the coupling between them. The multichannel equations are then solved subject to scattering boundary conditions

(3) $$\begin{array}{l} F_{nn^{\prime }}\left(\rho \right)=0\text{ as }\rho \to 0 \end{array}$$

(4) $$\begin{array}{l} F_{nn^{\prime }}\left(\rho \right)\sim \frac{1}{2ik_{n}}\left(\delta_{nn^{\prime }}\exp\left[-ik_{n}\rho \right]-S_{nn^{\prime }}^{J}\left(E\right)\exp\left[ik_{n}\rho \right]\right)\\ \text{ as }\rho \to \infty \end{array}$$

to yield the partial wave scattering matrix elements $S_{nn^{\prime }}^{J}\left(E\right)$ : these are the fundamental quantities for generating reactive transitions from an initial state *n* to a final state *n*′ as a function of total energy. For specific total energy, *E*, and total angular momentum quantum number, *J*, the computer code [Skouteris et al. (2000)] provides the reactive scattering matrix. The square moduli of the S-matrix elements

(5) $$\begin{array}{l} P_{nn^{\prime }}^{J}\left(E\right)=|S_{nn^{\prime }}^{J}\left(E\right){|}^{2} \end{array}$$

are state-to-state reaction probabilities yielding the products in the roto-vibrational state *n*′ starting from the reactants in the roto-vibrational state *n*.

**Figure 1:**
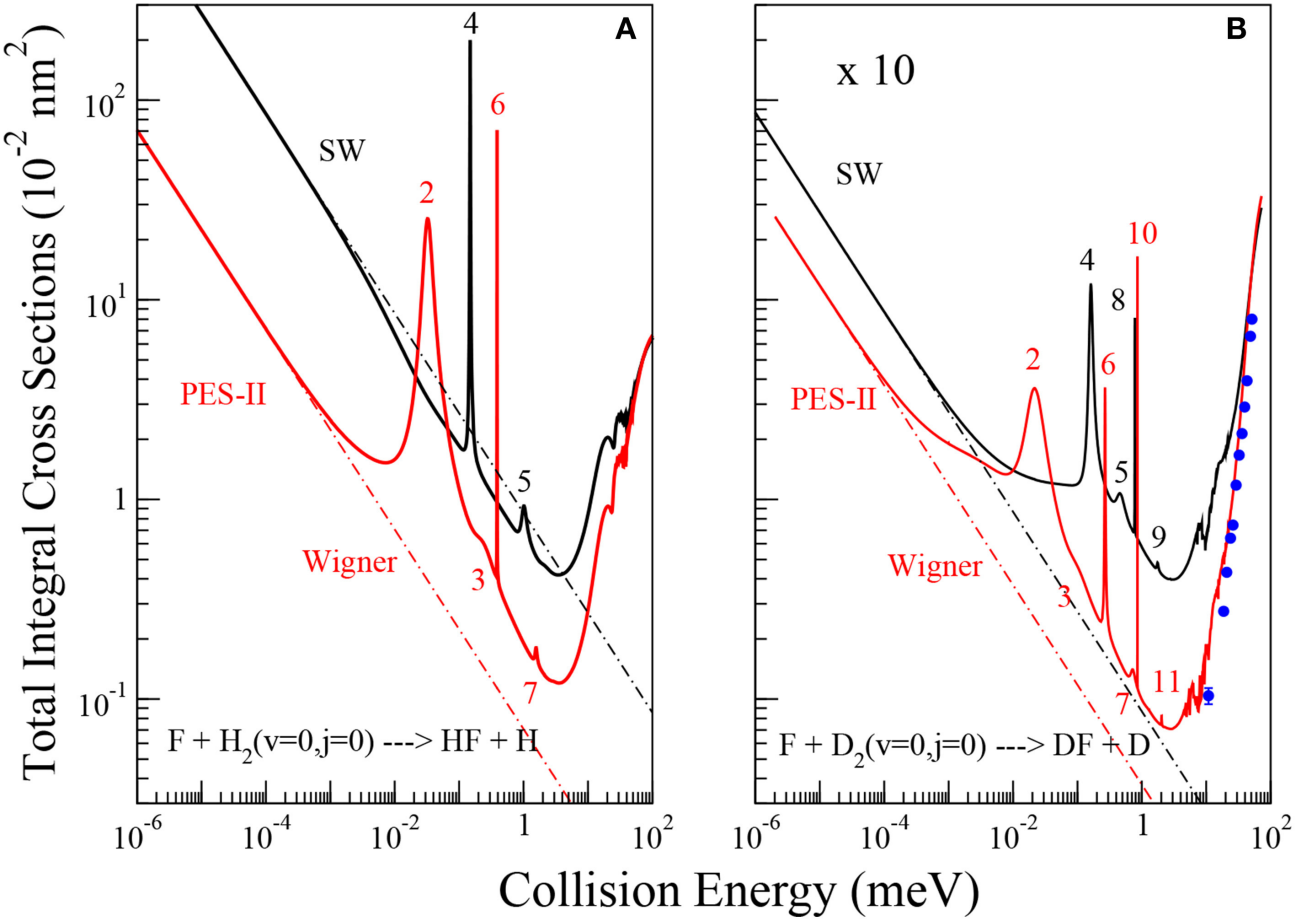
Energy dependence of the ics for the reactions of the F atom with H_2_, panel **(A)**, and D_2_, panel **(B)**, molecules in their ground roto-vibrational state. The solid black and red lines are the results using SW PES and PES-II, respectively. The dot-dashed lines show the fittings of numerically exact ics to the Wigner threshold law (Equation 8). A pattern of *J*-selected resonance peaks appears in the cold energy region. The value of the total angular momentum quantum number where the peaks show up in the reaction probabilities is indicated. Blue dots with error bars are the experimental values of reference (Che et al., 2007).

**Figure 2:**
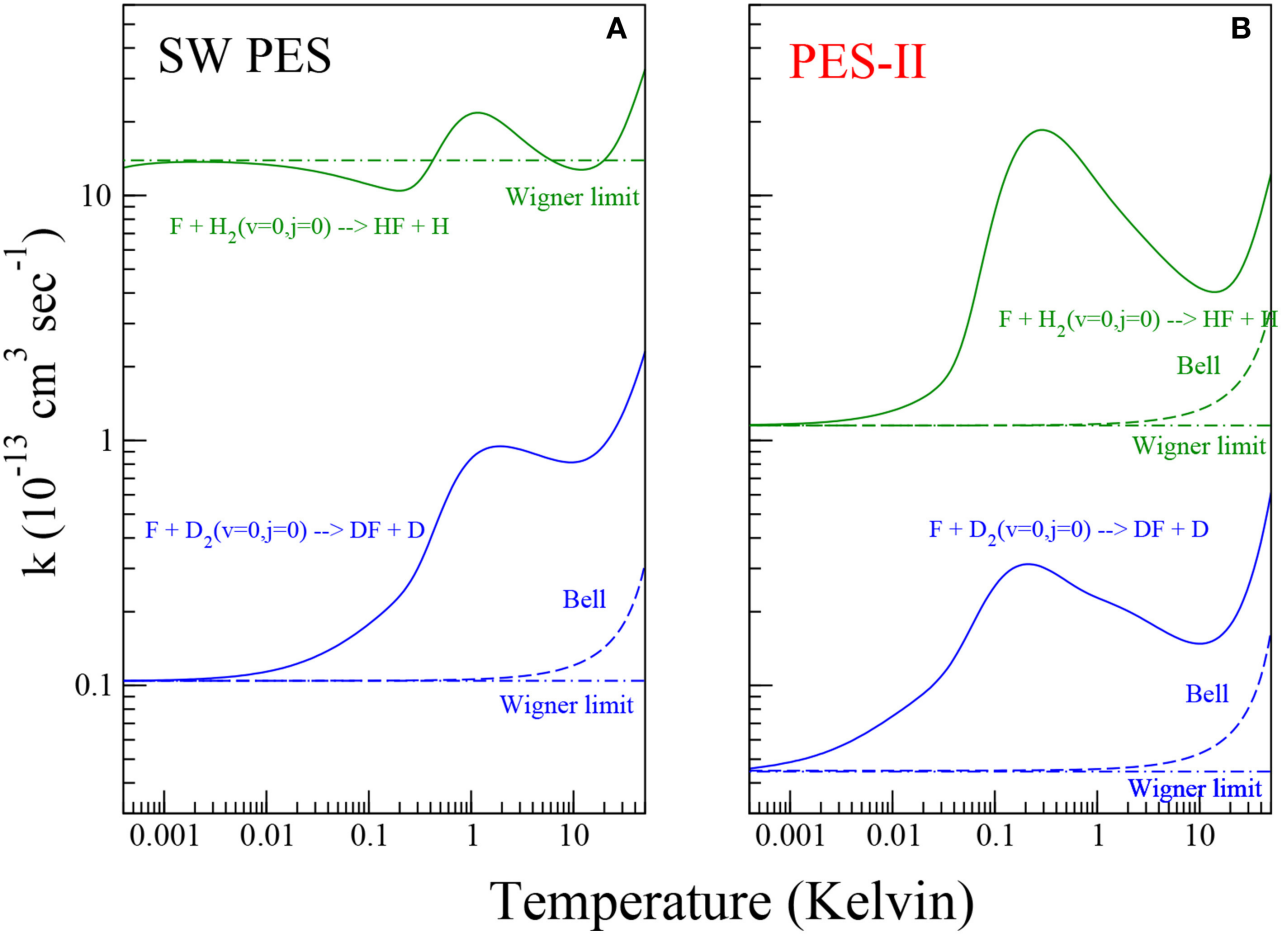
Rate constants for the reaction of the F atom with H_2_ (green color) and D_2_ (blue color) molecules on the SW PES [panel **(A)**] and PES-II [panel **(B)**]. The solid lines show the numerically exact results obtained by rigorous quantum reactive scattering calculations (Equation 7). The dashed lines depict the Bell approximation (Equation 11). The dot-dashed lines show the Wigner limit (Equation 10).

Quantum reactive scattering calculations serve to generate differential and integral cross sections as well as rate constants, the observable quantities in reaction dynamics and kinetics experiments. The comparison between experiments and theory supplies the most stringent test for the reliability of potential energy surfaces. Formulas used to calculate the cross sections and the rate constants shown in Figures 1, 2 are summarized in the following.

The initial state-selected total integral cross section at given values of the relative translational energy of reactants, *E*_*c*_, can be expressed as the sum of reaction probabilities

(6) $$\begin{array}{l} \sigma_{n}\left(E_{c}\right)=\frac{\pi }{g_{n}k_{n}^{2}}\underset{n^{\prime }}{\sum }\underset{J}{\sum }\left(2J+1\right)P_{nn^{\prime }}^{J}\left(E\right) \end{array}$$

over all the final states, *n*′, and the partial waves, *J*, accessible during the reaction, with *g*_*n*_ and *k*_*n*_ being the initial state degeneracy factor and wave vector, respectively. The dependence of the rate constant on temperature is calculated averaging σ_*n*_ over the Maxwell-Boltzmann distribution of the initial translational energy

(7) $$\begin{array}{l} k_{n}\left(T\right)={\left(\frac{2}{k_{B}T}\right)}^{3/2}\frac{1}{{\left(\pi m\right)}^{1/2}}\int_{0}^{\infty }E_{c}\sigma_{n}\left(E_{c}\right)\exp\left(-E_{c}/k_{B}T\right)\text{ d}E_{c} \end{array}$$

where *k*_*B*_ is the Boltzmann constant and *m* is the reduced mass of the reactants. The thermal rate constant is the sum over all the initial states accessible at a given temperature *T* weighted for the relative population of the state *n*, where *n* ≡ (*v, j*) stands for the vibrational and rotational quantum numbers *v, j* of the molecule.

Quantum scattering calculations on SW PES and PES-II have been carried out using a parallelized variant of the computer code (Skouteris et al., 2000) implementing the Enhanced Renormalized Numerov method (Colavecchia et al., 2003) for the integration of the hyperradial multichannel equations in Equation (2) above, see references (De Fazio, 2014; De Fazio et al., 2016) for more details. These changes make the code more efficient, especially at very low collision energies. The input parameters used in the production run are given in Tables S1, S2. After extracting the scattering matrix we calculate the total integral cross sections from Equation (6) and then perform the thermal averaging in Equation (7) to obtain the rate coefficients. These latter have been divided by the electronic partition function of the fluorine atom to account for its open-shell structure. As far as the temperature dependence of the electronic partition function is concerned, it has been properly taken into account in rate constant calculations. However, in the range of temperature investigated, it is essentially negligible (<0.001 %) and was not considered in the calculation of the tunneling correcting factor in Equation (12).

## 3. Results and Discussion

The effects of quantum mechanical tunneling and resonances on reaction cross sections and rate constants of the title reactions are discussed in the following.

### 3.1. Reaction Cross Sections

The total integral cross sections (ics) of the F + H_2_(*v* = 0;*j* = 0) → HF + H and F + D_2_(*v* = 0;*j* = 0) → DF + D chemical reactions have been calculated in a wide collision energy interval ranging from the ultra-cold region to above the reaction barrier (10^−6^- 10^2^ meV), as shown in Figure 1. In each panel, the results obtained using SW PES and PES-II are compared. In the right panel we also report the experimental values of reference (Che et al., 2007) with the relative error bars. The good agreement with PES-II results corroborates the reliability of this PES for describing the low collision energy dynamics of this system.

The ics have a very similar dependence on energy, with a minimum at about 4 meV and the appearance of narrow resonance patterns between 0.01 and 2 meV. However, the number of resonance peaks, their position and intensity vary markedly as the PES and the isotopic variant are changed. Broader resonance features also appear between 20 and 100 meV. Below 0.01 meV the ics increase smoothly with decreasing collision energy, quickly approaching the limiting behavior predicted by the Wigner's threshold law (Wigner, 1948). The broader peaks appearing above 20 meV have been analyzed in detail in previous papers (Castillo et al., 1996; Der Chao and Skodje, 2002; Aquilanti et al., 2004) which have provided a satisfactory description of the spectrum of metastable states of the F + H_2_ reaction. In particular, the oscillations observed have been interpreted as an interference effect between two of them: a resonance trapped in the transition state region and the other one supported by the van der Waals well of the exit channel (Cavalli and De Fazio, 2007, 2011; Sokolovski et al., 2007b). A clear explanation of the oscillatory pattern in the integral cross section has also been given in terms of Regge oscillations (Sokolovski et al., 2007a).

#### 3.1.1. The Cold Collision Regime

As far as the sharp resonance peaks in the cold energy regime are concerned, we have found that they are narrow isolated resonances affecting just a single partial wave. From the analysis of the reaction probabilities, we have been able to label each peak with the value of the total angular momentum quantum number, *J*, at which the resonance state appears. As shown in Figure 1, in most cases the sequences of the *J* values labeling the resonances are not regular and many values are missing. This observation suggests that the observed patterns do not originate from the rotational levels of a single metastable state. To support this hypothesis, we have calculated the vibrational energies of the triatomic van der Waals complex in the entrance channel at selected *J* values, see Table 1. For the calculation of the roto-vibrational energies we have used an adiabatic model. In brief, we have extended the *J* = 0 quasi-bound state calculations of Takayanagi and Kurosaki (1998) to larger values of *J*. The energies have been calculated by solving, at fixed *J*, a one-dimensional bound state problem in *R*, the intermolecular distance between the F atom and the H_2_/D_2_ molecule. The matrix elements of the Hamiltonian have been evaluated numerically in a basis of asymptotic diatomic roto-vibrational functions. More details of the calculations done will be given elsewhere.

**Table 1:** Roto-vibrational states of the three-atomic complexes F···H_2_ and F···D_2_ supported by the entrance channel van der Waals well on SW PES and PES-II.

		SW PES		PES-II	
***J***	***v***	***E*_*t*_/*eV***	**$E_{b}/cm^{-1}$**	***E*_*t*_/*eV***	**$E_{b}/cm^{-1}$**
F···*H*_2_					
0	0	0.2662	−15.7	0.2643	−31.4
0	1			0.2680	−1.5
1	0	0.2664	−13.9	0.2645	−29.6
1	1			0.2681	−0.7
2	0	0.2669	−10.3	0.2649	−26.1
3	0	0.2675	−5.1	0.2656	−20.9
4	0			0.2664	−14.2
5	0			0.2674	−6.1
F···*D*_2_					
0	0	0.1861	−32.5	0.1842	−48.2
0	1	0.1896	−4.2	0.1887	−11.8
0	2			0.1901	−0.4
1	0	0.1862	−31.3	0.1843	−47.1
1	1	0.1897	−3.5	0.1888	−11.1
1	2			0.1901	−0.1
2	0	0.1865	−29.0	0.1846	−44.9
2	1	0.1898	−2.1	0.1889	−9.6
3	0	0.1870	−25.5	0.1850	−41.7
3	1	0.1901	−0.1	0.1892	−7.5
4	0	0.1875	−21.0	0.1855	−37.5
4	1			0.1896	−4.7
5	0	0.1882	−15.5	0.1861	−32.3
5	1			0.1900	−1.4
6	0	0.1890	−9.0	0.1869	−26.1
7	0	0.1899	−1.7	0.1878	−19.0
8	0			0.1888	−11.1
9	0			0.1898	−2.5

**J* and *v* are the total angular momentum and vibrational quantum numbers, respectively. *E*_*t*_ and *E*_*b*_ denote the total and the bound state energies (the zero energy is located at the bottom of the reactant valley)*.

From the results of Table 1, we can see that the number of rotational levels for each vibrational state coincides, in all cases, with the lowest value of *J* in each rotational progression shown in Figure 1. The *J*- selected peaks appearing in the ics are therefore due to shape resonances trapped in the van der Waals well-located in the entrance channel of the reaction: increasing the centrifugal energy the (quasi-)bound vibrational states escape from the well affecting the reactivity of the two successive partial waves. Thus, the number of bound vibrational states of the van der Waals complex determines the number of *J* progressions observed in each panel of Figure 1. These conclusions also explain why the number of peaks is different for each isotopic variant studied as well as for the two PESs: the number of resonance states is greater the stronger the long-range forces and the lower the vibrational frequency of the triatomic complex. The deeper and wider van der Waals well of PES-II supports more states that of SW PES: for example in the H_2_ case two vibrational states are bound in PES-II and just one in SW PES. Moreover, the vibrational frequency decreases with the increase of the isotopic mass so that the number of bound states is larger for the F···D_2_ van der Waals complex: three vibrational states are bound in PES-II and two in SW PES. Finally we note that these resonance features were also found in reference (Lique et al., 2011) but were erroneously attributed to Feshbach resonances in the exit van der Waals well.

#### 3.1.2. The Ultra-Cold Limit

In the ultra-cold energy range (below 0.001 meV) only *J* = 0 contributes to the partial wave expansion in Equation (6), so that the dependence of ics on collision energy follows the limiting behavior well-described by the Wigner's threshold law (Wigner, 1948):

(8) $$\begin{array}{l} \sigma_{W}=\frac{4\pi \hbar \beta }{k} \end{array}$$

where σ_*W*_ denotes the ics in the Wigner regime, β is the imaginary part of the scattering length [Balakrishnan et al. (1997)] and $k=\sqrt{2mE_{c}}/\hbar$ is the reactants' wave number. Fitting the ics shown in Figure 1 to Equation (8) we have obtained the values of the imaginary scattering lengths, see Table 2. Comparing the β values reported in the table, we see that the dispersion forces and isotopic substitutions influence both the collision energy of the onset of the Wigner regime and the magnitude of β. Because of the stronger long-range interaction potential of PES-II with respect to that of SW PES, the Wigner regime manifests itself to lower collision energy (approximately at 10^−4^ meV for PES-II, as shown in Figure 1). The larger barrier width of PES-II reduces significantly the β values. Also, we can note that lighter isotopic substitution yields larger β values. Again, for the H_2_ case the Wigner regime is encountered at higher energies although the effect is of minor entity than changing the entrance channel of the PES. However, the large differences found in the F + H_2_ case, where β changes by more than one order of magnitude, suggest that tunneling cannot be the only reason of this different behavior between the PESs.

**Table 2:** Values of the imaginary scattering length, β, in atomic units.

	**SW PES**	**PES-II**
F + H_2_	1.201 × 10^−1^	9.933 × 10^−3^
F + D_2_	1.644 × 10^−3^	7.064 × 10^−4^

In reference Bodo et al. (2004), analyzing elastic and reactive *J*=0 reaction probabilities obtained with the SW PES, the authors were able to obtain the real and imaginary parts of the scattering length. The values obtained were *a* = (−2.0−6.3 × 10^−2^*i*)Å and *a* = (3.95−8.6 × 10^−4^*i*)Å for the FH_2_ and FD_2_ systems, respectively. Comparing with the values reported in Table 2 we can see that the imaginary part of the scattering lengths is in perfect agreement with our results. The negative value obtained for the real part of the scattering length for H_2_ is attributed to the presence of a virtual state associated with the van der Waals well in the entrance channel and located around 0.3 meV below the reactive threshold. Fingerprints of this effect can also be noted in the left panel of Figure 1, where the SW PES curve shows up a faster increase around to 0.01 meV before reaching the Wigner regime. Additional evidences will be given later. For the F+D_2_ reaction, the positive value of the real part of the scattering length suggests that the scattering may be influenced by a resonance state. However, no structure has been detected in the reactive observables.

### 3.2. Reaction Rates at Low and Ultra-Low Temperatures

In Figure 2, the rate constants for the production of HF and DF molecules in the temperature range between 0.5 mK and 50 K are shown on log-log plots. Panels (A) and (B) show the results of quantum scattering calculations using SW PES and PES-II, respectively. As we can see from the figure, the kinetics of the two reactions studied is markedly influenced by resonance and tunnel quantum effects. The strong *J*-selected resonance features appearing in the cold collision domain, see Figure 1, survive to the Boltzmann averaging, see Equation (7) in section 2, giving a maximum at about 1 K. At lower temperature the rates decrease until they reach the Wigner regime at about 1 mK becoming independent of temperature. However, we can note that the SW PES curve in the left panel of Figure 2 behaves differently from the other, showing a minimum around 0.1 K that is not present in the other curves. This is again a manifestation of the virtual state effect discussed in the previous section. As discussed in reference (Simbotin and Côté, 2015), in the presence of a virtual or a resonance state near to the reactive threshold, in the so called Near Threshold Resonance (NTR) regime, the rate constant scales with temperature as 1/*T* before reaching the Wigner limiting value. Depending on the proximity of the virtual/bound state to the channel threshold, the reaction rate may increase by orders of magnitude. In this case (see also Simbotin and Côté, 2015) the virtual state is not so near, so that the 1/*T* behavior is just barely apparent.

In the plots of Figure 2 we can clearly distinguish three different regions as the temperature increases. Up to 1 mK the only pathway for the reaction is the tunneling through the barrier eventually enhanced by the virtual state effect. In the region intermediate between 1 mk and 10 K, the reactivity is additionally affected by the shape resonances in the entrance channel. Above 10 K, the scattering resonances trapped in the transition state and in the exit channel as well as the thermal activation mechanism cause a further increase in the rate coefficients.

In the so-called classical region, where the reactivity is dominated by the thermal activation pathway, the dependence of rate constants with temperature is described by the Arrhenius' law, predicting an exponential decrease of the reaction rate constants against reciprocal temperature

(9) $$\begin{array}{l} k=A\exp\left(-E_{a}/RT\right) \end{array}$$

where *E*_*a*_ denotes the activation energy and *A* is the pre-exponential factor. When the reaction also proceeds through the tunneling mechanism, the rate constants are larger than this law would predict and concave Arrhenius plots are observed. For chemical reactions where the exchange of the light hydrogen atom represents the rate determining step, the deviations can be pronounced: they depend on the tunnel effect. As the temperature tends toward zero (below 1 mK), according to the Wigner law (Wigner, 1948), the rate constants

(10) $$\begin{array}{l} k_{W}=\frac{4\pi \hbar \beta }{m} \end{array}$$

become temperature independent. An extra factor of 1/2 must be inserted to account for the open-shell structure of the fluorine atom (Aquilanti et al., 2005b). The temperature, *T*_*c*_, at which the low- and high-temperature limits in Equations (9, 10) cross is defined as the cross-over temperature (Hänggi et al., 1990) delimiting the deep (*T* < *T*_*c*_) and moderate (*T*_*c*_ ≲ *T*≲ 2 *T*_*c*_) tunneling regimes. According to Christov (1997), at the temperature *T* = 2*T*_*c*_ the tunneling and over-barrier mechanisms play the same role. In 1935 Bell found a formula bridging the low- and high-temperature limits of the rate coefficient

(11) $$\begin{array}{l} k_{Q}=AQ\exp\left(-V^{*}/RT\right) \end{array}$$

where V^*^ is the reaction barrier height and

(12) $$\begin{array}{l} Q=\frac{T-T_{c}\exp\left[V^{*}\left(1/RT-1/RT_{c}\right)\right]}{T-T_{c}} \end{array}$$

is a tunneling correcting factor (Bell, 1980). The above expression, written so to emphasize its dependence on the cross-over temperature, holds in the case of a truncated parabolic barrier, for more details see Cavalli et al. (2014). Toward the absolute zero, Equation (11) leads correctly to a non-vanishing and temperature independent expression while at high temperature $E_{a}=V^{*}$ and the Arrhenius rate expression (9) is recovered. For the F+H_2_ reaction, the temperature dependence of the activation energy has been calculated using a phenomenological approach (Aquilanti et al., 2012) in the range 10–350 K.

The quantitative enhancement that resonances have on the tunneling can be defined in terms of a coefficient

(13) $$\begin{array}{l} \gamma =\frac{k}{k_{Q}} \end{array}$$

that we take to be the ratio of the numerically exact rate constant *k*, obtained from rigorous quantum scattering calculations, to the tunneling corrected rate constants *k*_*Q*_, calculated from Equation (11) using the Arrhenius parameters *A* and V^*^ reported in Persky and Kornweitz (1997). The values of *T*_*c*_ have been obtained by imposing the limit for T = 0 of Equation (11) equal to Equation (10), namely:

(14) $$\begin{array}{l} T_{c}=\frac{-V^{*}}{R\ln\left[\left(4\pi \hbar \beta \right)/\left(mA\right)\right]}. \end{array}$$

**Figure 3:**
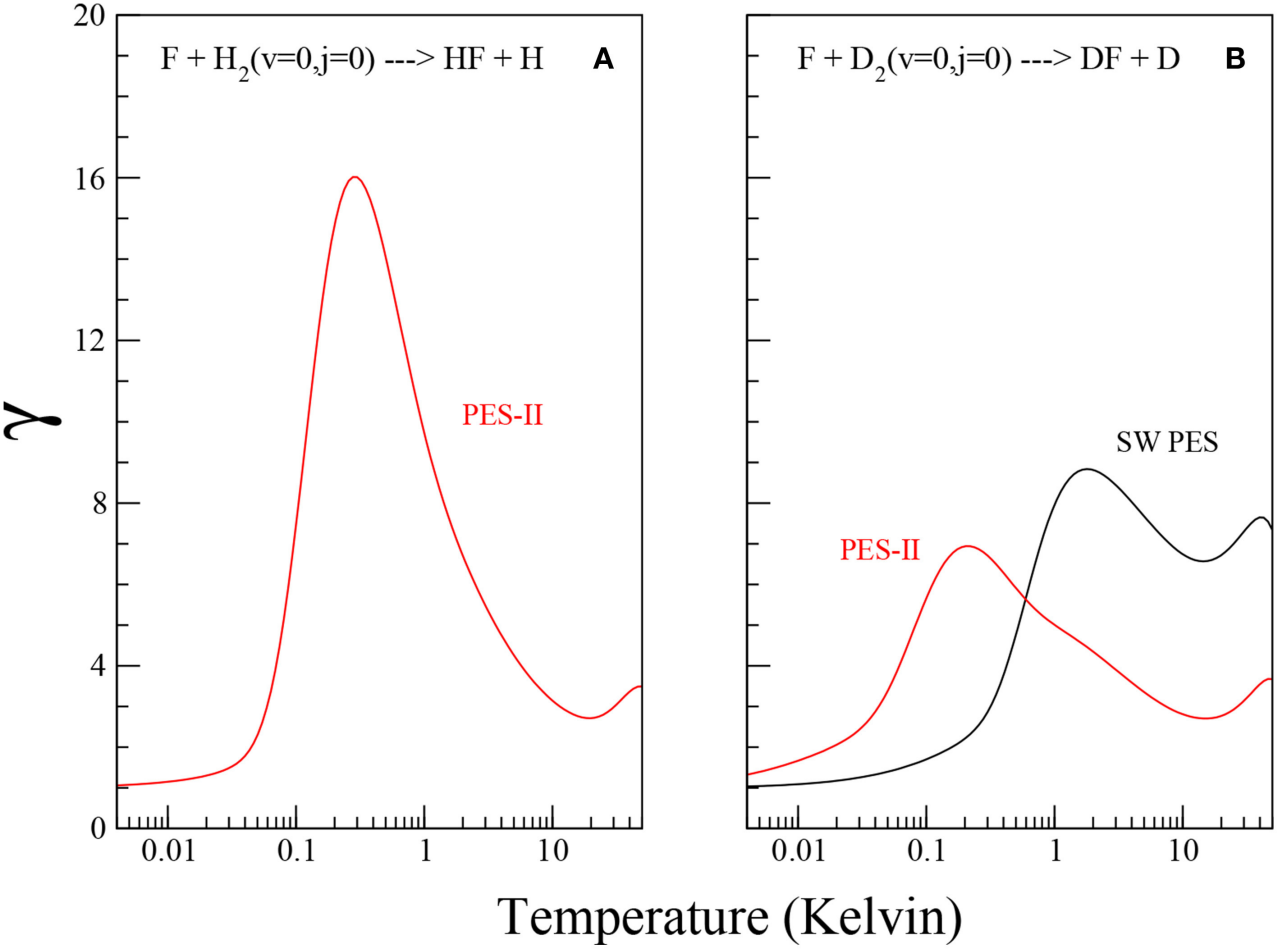
Plots of the γ coefficient as defined in Equation (13) for the F+H_2_ and F+D_2_ reactions, panels **(A,B)**, respectively, on the SW PES (black solid lines) and PES-II (red solid lines).

Using the values of the imaginary scattering length, β, reported in Table 2, we obtain *T*_*c*_ ≃ 74.4 K and 67.7 K for F + D_2_ SW PES and PES-II, respectively and *T*_*c*_ ≃ 72.9 K for F + H_2_ PES-II. Note that Equation (14) does not hold in presence of virtual state effects because the model used to obtain Equation (11) does not take it into account. For this reason we can not obtain *T*_*c*_ and γ in the F+H_2_ SW PES case. The temperature dependence of the γ coefficient is shown in Figure 3. Note that γ is always lager than one, as it should be. The pronounced peak at very low temperatures is the overall contribution of the *J*-selected shape resonances, while the shallow maximum above 10 K is due to the presence of a transition state resonance (Cavalli and De Fazio, 2007).

#### 3.2.1. Kinetic Isotope Effect

**Figure 4:**
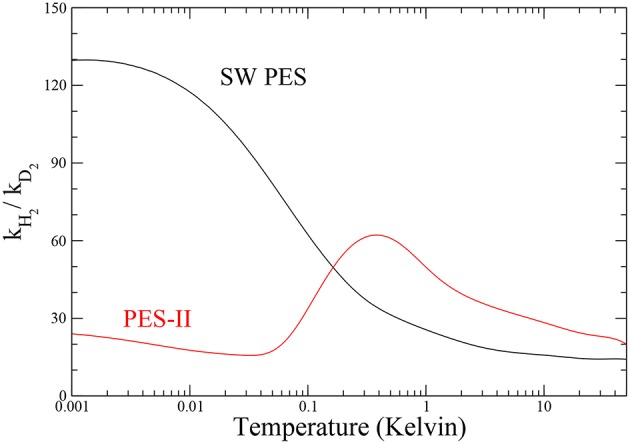
Temperature dependence of the inter-molecular kinetic isotope effect for the two potential energy surfaces investigated.

The ratio between the rates of the lighter and the heavier isotopic combinations is a measure of the inter-molecular kinetic isotope effect (KIE) (Steckler et al., 1985). The temperature dependence of KIE is shown in Figure 4. The rate constant of the F + H_2_ reaction increases drastically as temperature decreases, so that in the ultra-cold regime the fluorine atom is about one hundred times more reactive with H_2_ than with D_2_ on SW PES. We note that this effect is about two orders of magnitude larger than the semi-classical limit (Bigeleisen, 1949), which applies above room temperature, where the tunnel effect is negligible and the main source of KIE comes from the difference among the reactants' zero point energies, the so-called primary KIE. At low temperatures (until 1 K), the features are markedly influenced by the tunnel effect giving an exponential enhancement (Bell, 1980) more evident in PES-II where the barrier is thicker. Below 1 K the KIE behavior in two PESs is very different: a typical sigmoid curve is observed on SW PES, while the resonance enhancement of the rates give rise to a maximum observed at about 1 K in the case of PES-II. This big difference is of course due to the presence of the virtual state effect that enhances selectively the F + H_2_ rate of the SW PES curve, hiding also the smaller resonance KIE effect. Below 1 mK the rates are temperature independent and therefore also is the KIE.

## 4. Conclusions

The reactivity of the F atom with the H_2_ and D_2_ molecules in their ground roto-vibrational state has been investigated in the quantum mechanical low-temperature regime (0.5 mK–50 K), where because of the prominent role of tunneling and resonances, the rates show pronounced deviations from the Arrhenius law before they become independent of temperature near absolute zero according to the Wigner law. The latter, unlike the Arrhenius' law, predicts that chemical reaction rates do not tend to zero when collision energies become vanishingly small. Total integral cross sections and rate constants have been calculated by numerically exact quantum reactive scattering calculations carried out on two potential energy surfaces, the widely used SW PES and a variant of it referred to as PES-II, differing in the description of the van der Waals well in the reactants' channel. The results have shown that the small changes in the entrance channel interaction and the isotopic substitution lead to the enhancement of cross sections and rate coefficients by many orders of magnitude and induce an unexpected dependence of the intermolecular kinetic isotope effect on temperature.

A quantitative assessment of the extent of the roles of tunneling and resonances, as well as that of the energy region where these quantum effects show up, has been provided. We have shown that the wide interval of collision energies and temperatures analyzed can be adequately divided into smaller domains within which the reactivity is differently influenced by the different quantum mechanical effects: at ultra-cold energies, the title reaction and its isotopic variant occur only via the tunneling pathway possibly enhanced by virtual state effects; in the cold collision regime, cross sections and rate coefficients are additionally affected by shape resonances in the entrance channel of the potential energy surface, that enhance the contribution of the tunnel effect; finally, as the collision energy gets closer to the height of the classical barrier, the tunneling enhanced by a resonance state trapped in the transition state region of the potential energy surface competes with the over-barrier mechanism.

The ultra-cold results obtained with the SW PES corroborate previous studies (Bodo et al., 2004; Simbotin and Côté, 2015) made with the same PES, namely that a virtual state in the reactants' van der Waals well deeply affects the ultra-cold reactivity of the F+H_2_ reaction. However, no evidence of this effect is found in the PES-II calculations. The large difference in the KIE behavior between the two PES suggests that this is likely the most sensitive observable to emphasize these features.

## Author Contributions

All authors listed have contributed equally to the work, and approved it for publication.

### Conflict of Interest Statement

The authors declare that the research was conducted in the absence of any commercial or financial relationships that could be construed as a potential conflict of interest.
