# Supplementary material for: Quantum Dynamics and Kinetics of the F + H2 and F + D2 Reactions at Low and Ultra-Low Temperatures
Source: Front Chem. 2019 May 14;7:328. doi: 10.3389/fchem.2019.00328 (PMC6527900; doi:10.3389/fchem.2019.00328)
Supplement: Supplementary file 1 [file Data_Sheet_1.pdf]

frontiers

Supplementary Material: Quantum dynamics and kinetics of the $F+H_2$ and $F+D_2$ reactions at low and ultra-low temperatures

1 SUPPLEMENTARY TABLES AND FIGURES

The input data for the $F+H_2$ and $F+D_2$ quantum scattering calculations on the SW potential energy surface and PES II are given in Table S1 and Table S2.

Table S1. Input parameters used in the production run of the $F+H_2$ reaction.

E/meV	J	Kmax	j	emax	rmax	mtr	prod	dE/meV
				SW PES				
96-145	20	6	18	2.3	10	100	100	0.5
46-95	17	5	18	2.3	15	150	100	0.5
22-42	13	3	18	3.0	15	150	100	0.2
2.2-21.9	11	3	18	2.5	20	200	99	0.2
1.2-2.2	7	3	18	2.3	20	235	100	1.0(-2)
0.2-1.2	5	3	18	2.3	25	300	104	1.0(-2)
risuk								
1.4(-1)-1.6(-1)	4	3	20	3.3	25	700	18	4.0(-4)
1.1(-2)-1.4(-1)	4	2	16	3.0	30	300	128	1.0(-3)
1.0(-3)-1.1(-2)	2	1	16	2.5	28	320	97	1.0(-4)
0.6(-4)-1.0(-3)	1	1	16	2.8	35	400	100	1.0(-5)
0.8(-5)-0.6(-4)	0	0	16	2.8	35	400	50	1.0(-6)
				PES II				
96-145	21	6	18	2.3	15	150	100	0.05
46-95	19	5	18	2.3	20	200	100	0.05
22-42	13	3	24	3.0	18	180	100	0.02
2.1-21.9	11	2	24	3.0	25	250	100	0.02
1.1-2.1	7	2	16	2.5	30	300	100	1.0(-2)
0.3-1.1	6	1	16	2.5	40	400	84	1.0(-2)
risuk	6						37	1.0(-3/-4)
6.9(-2)-2.7(-1)	4	1	16	2.5	50	500	100	2.0(-3)
1.1(-2)-5.3(-2)	3	2	80	3.8	80	1000	86	0.5(-3)
1.0(-3)-1.1(-2)	2	1	18	3.0	100	1000	98	1.0(-4)
1.0(-4)-1.0(-3)	2	1	16	2.5	140	1400	94	1.0(-5)
1.0(-5)-1.0(-4)	1	0	16	2.5	180	1800	91	1.0(-6)
0.4(-6)-1.0(-5)	0	0	16	2.5	220	2200	100	1.0(-7)

For explanation of input parameters see Skouteris et al. (2000); De Fazio (2014); De Fazio et al. (2016).

Table S2. Input parameters used in the production run for the F+D_2 reaction.

E/meV	J	K_{max}	j	e_{max}	r_{max}	mtr	prod	dE/meV
				SW PES				
39.9-72.1	19	4	25	3.0	15	150	130	0.25
22.4-39.6	15	3	28	3.0	20	275	70	0.25
2.3-22.2	14	2	20	2.5	20	350	200	0.01
0.22-2.19	11	2	20	2.5	35	500	198	1.0(-2)
2.0(-2) 2.2(-1)	6	1	20	2.5	70	1030	198	1.0(-3)
2.2(-3)-2.2(-2)	2	2	34	2.8	100	1325	198	1.0(-4)
2.2(-4)-2.2(-3)	2	1	25	3.0	130	1750	198	1.0(-5)
2.2(-5)-2.2(-4)	1	1	22	3.0	150	2025	198	1.0(-6)
2.0(-6)-2.2(-5)	0	0	22	3.0	180	2435	200	1.0(-7)
				PES II				
39.9-72.1	19	4	25	3.0	15	150	130	0.25
22.4-39.6	15	3	28	3.0	20	275	70	0.25
2.3-22.2	14	2	20	2.5	20	350	200	0.01
0.22-2.19	11	2	20	2.5	35	500	198	1.0(-2)
2.0(-2) 2.2(-1)	6	1	20	2.5	70	1030	198	1.0(-3)
2.2(-3)-2.2(-2)	2	2	34	2.8	100	1325	198	1.0(-4)
2.2(-4)-2.2(-3)	2	1	25	3.0	130	1750	198	1.0(-5)
2.2(-5)-2.2(-4)	1	1	22	3.0	150	2025	198	1.0(-6)
2.0(-6)-2.2(-5)	0	0	22	3.0	180	2435	200	1.0(-7)

For explanation of input parameters see Skouteris et al. (2000); De Fazio (2014); De Fazio et al. (2016).

REFERENCES

Skouteris, D., Castillo, J., and Manolopoulos, D. (2000). Abc: a quantum reactive scattering program. *Computer Physics Communications* 133, 128–135

De Fazio, D. (2014). The H+HeH$^+$ $\rightarrow$ He + H_2^+reaction from the ultra-cold regime to the three-body breakup: exact quantum mechanical integral cross sections and rate constants. *Physical Chemistry Chemical Physics* 16, 11662–11672

De Fazio, D., Cavalli, S., and Aquilanti, V. (2016). Benchmark quantum mechanical calculations of vibrationally resolved cross sections and rate constants on ab initio potential energy surfaces for the F+HD reaction: Comparisons with experiments. *The Journal of Physical Chemistry A* 120, 5288–5299
